# A novel Respiratory Health Score (RHS) supports a role of acute lung damage and pig breed in the course of an *Actinobacillus pleuropneumoniae *infection

**DOI:** 10.1186/1746-6148-5-14

**Published:** 2009-04-21

**Authors:** Doris Hoeltig, Isabel Hennig-Pauka, Kerstin Thies, Thomas Rehm, Martin Beyerbach, Katrin Strutzberg-Minder, Gerald F Gerlach, Karl-Heinz Waldmann, FUGATO-consortium IRAS

**Affiliations:** 1 Clinic of Swine and Small Ruminants, Forensic Medicine and Ambulatory Service, University of Veterinary Medicine Hannover, Bischofsholer Damm 15, 30173 Hannover, Germany; 2 Institute for Microbiology, Department of Infectious Diseases, University of Veterinary Medicine Hannover, Bischofsholer Damm 15, 30173 Hannover, Germany; 3 Department of Biometry, Epidemiology and Information Processing, University of Veterinary Medicine Hannover, Bünteweg 2, 30559 Hannover, Germany; 4 IVD GmbH, Heisterbergallee 12, 30435 Hannover, Germany

## Abstract

**Background:**

Bacterial lung infections are a major cause of economic losses in the pig industry; they are responsible for approximately 50% of the antibiotics used in pigs and, therefore, also present an increasing concern to consumer protection agencies. In response to this changing market we investigated the feasibility of an old approach aimed at the breeding selection of more resistant pigs. As a first step in this direction we applied a new respiratory health score system to study the susceptibility of four different pig breeding lines (German Landrace, Piétrain, Hampshire, Large White) towards the respiratory tract pathogen *Actinobacillus *(*A*.) *pleuropneumoniae*.

**Results:**

A controlled experimental aerosol infection with an *A. pleuropneumoniae *serotype 7 isolate was performed using 106 weaning pigs of defined breeding lines from the breeds German Landrace, Piétrain, Hamphire, and Large White. Pigs were clinically assessed on days 4 and 20 post infection following a novel scoring system, the Respiratory Health Score (RHS), which combines clinical, sonographic and radiographic examination results. The ranking on day 4 was significantly correlated with the ranking based on the pathomorphological Lung Lesion Score (LLS; Spearman Rank Correlation Coefficient of 0.86 [p < 0.0001]). Based on their RHS pigs were assigned to the different quartiles independent of the breeding line. The RHS-based rankings of pigs on day 4 and on day 20 were highly correlated (Spearman Rank Correlation Coefficient of 0.82 [p < 0.0001]) independent of the breeding line. Pigs of the Hampshire line were predominantly found in the lowest scoring quartile (47.6%) and absent in the highest scoring quartile. In contrast, pigs of the German Landrace and Piétrain breeding lines were predominantly found in the highest scoring quartile (32.3% and 35.7%, respectively).

**Conclusion:**

These results demonstrate that the RHS obtained from live pigs shows a highly significant correlation to the lung lesion score considered as a "gold standard". The correlation of the ranking at days 4 and 20 post infection implies that the course of disease is highly dependent on the acute lung damage. The different severity of signs among the tested pig breeding lines clearly suggests a genetic difference in the susceptibility of pigs to *A. pleuropneumoniae *infection.

## Background

Bacterial respiratory diseases caused by *Mycoplasma hyopneumoniae, Actinobacillus pleuropneumoniae*, and *Haemophilus parasuis *are of major importance in the pig industry [1-3]. They cause severe economic losses due to premature deaths and reduced daily weight gains [4]. Currently, productivity in infected herds is improved by antibiotic treatment and by vaccination regiments. However, vaccination efficacy is hampered by limited cross-serovar protection [5,6], and treatment is impeded by the increasing occurrence of resistant strains [7]. In addition, in recent years the interest of consumers in quality assurance along the pork production chain has greatly heightened. Thus, today there is a strongly increasing demand for pork obtained from healthy animals not subjected to treatment during the fattening period [8,9].

In response to this changing market an old approach aimed at the breeding selection of more resistant pigs has gained renewed attention. In the past it could be shown that breed is an important factor influencing the baseline immune traits and, therefore, the response to various stressors or infectious challenges [10]. For example, the susceptibility of pigs towards *E. coli *K88 which was identified to be of monogenic origin has been successfully suppressed by a targeted breeding approach [11,12]. More recently, Reiner et al. showed that different breeds have distinct susceptibilities towards Pseudorabies virus [13] and *Sarcocystis miescheriana *[14]; QTL-mapping revealed that increased resistance to these pathogens is a complex trait. Also in the field of avian [15], ovine [16] and bovine diseases [17] natural resistance to different pathogens could be ascertained and used for the selection of a breeding population with increased resistance.

Concerning respiratory diseases of pigs a number of studies dealing with breed-dependent susceptibility towards viral infection (PCV-2, PRRSV) have been performed [18-22]. All of these studies provide strong evidence for genetic factors accountable for differences in susceptibility towards viral lung infections. In addition, a number of studies implied that there also is an influence of breed on the mortality of pigs suffering from bacterial lung infection [23-26].

The availability of increasing molecular information and new technologies such as i) a draft sequence of the porcine genome, ii) whole genome expression arrays, and iii) a porcine single nucleotide polymorphism (SNP) chip with 50.000 SNP now potentially facilitate the investigation of complex traits. As a first step in this direction we investigated the susceptibility of four different pig breeding lines (German Landrace, Piétrain, Hampshire, Large White) to infection with *A. pleuropneumoniae*. In order to minimize environmentally-induced variability, all pigs originated from the same herd and were subjected to experimental aerosol infection in a well-established *A. pleuropneumoniae *model [27-30]. All animals were phenotyped using an elaborate clinical investigation scheme including sonography and radiography before infection as well as on days 4 and 20 post infection [31], and a clinical assessment system, the Respiratory Health Score (RHS) was developed. Including all clinical investigation results this RHS facilitates an exact determination of the lung status in the living pig. Therefore, unlike the Lung Lesion Score (LLS), which is used as gold-standard for lung investigations until now [32], the RHS allows the comparison of the lung status at different time points after infection. The animals were consecutively ranked according to disease severity in acute (day 4) and chronic infection (day 20). Subsequently, the ranking of animals on both days and the number of animals from different breeding lines in the ranking quartiles were compared.

## Results

### Development and validation of the Respiratory Health Score (RHS)

Upon infection the severity of clinical symptoms ranged from no symptoms to death. Clinical symptoms were typical for *A. pleuropneumoniae *infection with body temperatures up to 41.9°C, dyspnoea, coughing and apathy; likewise, ultrasonographic and radiographic results with comet tail artefacts, interruption of the pleural line, liver-like parenchyma texture, abscesses, bronchography, and increasing lung opacity were typical for porcine pleuropneumonia.

**Figure 1:**
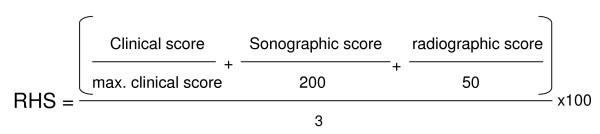
**Algorithm for calculating the Respiratory Health Score (RHS)**. Clinical, sonographic and radiographic scores were normalized by division with the score causing death in an animal, added, divided by three and multiplied by 100 in order to get a value in percent. The RHS has a possible range from 0 to 100%. The maximum clinical score on days 4 and 20 is 20 and 100, respectively.

**Figure 2:**
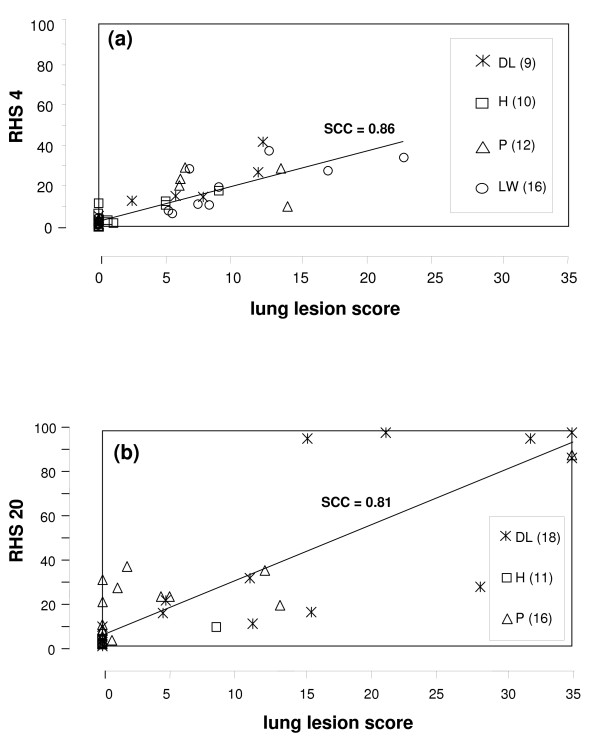
**Correlation of RHS and lung lesion score on day 4 (a), and on day 20 post infection (b)**. The Spearman Rank Correlation coefficient (SCC) was calculated using 47 (a) and 45 pigs (b), respectively, and was highly significant (p < 0.0001) for both time points. The number of pigs with a LLS of 0 was 21 (a) and 23 (b), respectively. DL = German Landrace, H = Hampshire, P = Piétrain, LW = Large White; the number in parenthesis indicate the number of pigs of each breeding line.

Of the 106 pigs challenged 103 pigs entered the study; all these pigs had a RHS of zero at day 7 pre-infection and tested negative for *A. pleuropneumoniae*, *Mycoplasma hyopneumoniae*, *Influenca-A-Virus *and *PRRSV *by antibody ELISA. Due to severe clinical symptoms 11 pigs died or were euthanized outside of days 4 and 20 and, therefore, could not be included in the subsequent ranking. Of the remaining 92 pigs 47 were sacrificed on day 4 and 45 on day 20 post infection, respectively. For each of these pigs a daily clinical score was determined and, together with the results of the ultrasonographic and radiographic lung examination, was used to calculate the RHS (Fig. 1). In addition, at necropsy the LLS was determined for each of these pigs. Pigs were then ranked based on RHS and LLS; the Spearman Rank Correlation Coefficient comparing both ranking scores was determined to be 0.86 and 0.81 (p < 0.0001) on day 4 (Fig. 2a) and on day 20 (Fig. 2b), respectively.

### Comparative ranking of pigs on days 4 and 20 post infection

**Figure 3:**
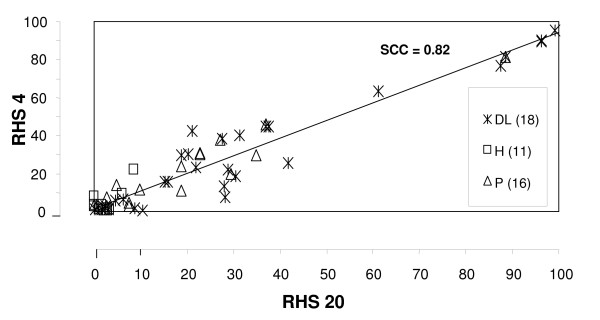
**Correlation of RHS on days 4 and 20 post infection**. The Spearman Rank Correlation coefficient (SCC) was calculated to using 45 pigs and was highly significant (p < 0.0001). DL = German Landrace, H = Hampshire, P = Piétrain, LW = Large White; the number in parenthesis indicate the number of pigs of each breeding line.

The RHS facilitated a comparative ranking of pigs on days 4 and 20 post infection, and this was performed for 45 pigs sacrificed on day 20 post infection (Fig. 3). A close correlation (Spearman rank correlation coefficient of 0.82) was observed and was found to be highly significant (p < 0.0001; Fig. 3).

### Susceptibility of different breeding lines upon infection with *A. pleuropneumoniae*

Eleven of 38 German Landrace pigs died or were euthanized due to severe clinical symptoms before day 4 or between day 4 and day 20. All pigs of the other breeding lines survived. The RHS-based ranking of all surviving pigs was performed on day 4 and 20 post infection. In order to assess the relative disease severity within the different breeding lines ranking quartiles were formed (Table 1), and the distribution of the different breeding lines within these quartiles was ascertained (Tables 2, 3). It was apparent that pigs of the Hampshire breeding line were clearly less susceptible to an *A. pleuropneumoniae *infection showing no mortality and no animals in the 4^th ^ranking quartile at both day 4 and day 20 post infection. The other three breeding lines showed no clear differences with respect to the RHS; thus in all lines at least 50% of the pigs were ranked in the 3^rd ^or 4^th ^quartile. When considering mortality in addition to the RHS, pigs of the German Landrace breeding line appeared to be most susceptible to *A. pleuropneumoniae *infection.

**Table 1:** Range of RHS-points within the ranking quartiles at 4 and 20 days post infection

	day 4 p. inf (n = 96)	day 20 p. inf. (n = 45)
1^st ^quartile (no symptoms)	0 – 2.1	0.21 – 3.33

2^nd ^quartile (mild symptoms)	2.11 – 10.7	3.34 – 18.8

3^rd ^quartile (moderate symptoms)	10.71 – 27.5	18.81 – 34.91

4^th ^quartile (severe symptoms)	27.51 – 95.61	34.92 – 99.12

**Table 2:** RHS-based distribution of pigs from different breeding lines into ranking quartiles on day 4 post infection

	1^st ^quartile^1)^	2^nd ^quartile^2)^	3^rd ^quartile^3)^	4^th ^quartile^4)^
German Landrace^5) ^n = 27	16.1%	29.0%	22.7%	32.2%

Hampshire n = 21	47.6%	28.6%	23.8%	0%

Piétrain n = 28	14.3%	28.6%	21.4%	35.7%

Large White n = 16	37.5%	12.5%	25.0%	25.0%

^1) ^RHS of 0 to 2.1; ^2) ^RHS of 2.11 to 10.7; ^3) ^RHS of 10.71 to 27.5; ^4) ^RHS of 27.51 to 95.61; ^5) ^Eleven German Landrace pigs died or were euthanized outside of days 4 and 20 and, therefore, were not included.

**Table 3:** RHS-based distribution of pigs from different breeding lines into ranking quartiles on on day 20 post infection

	1^st ^quartile^1)^	2^nd ^quartile^2)^	3^rd ^quartile^3)^	4^th ^quartile^4)^
German Landrace^5) ^n = 18	21.1%	10.5%	31.6%	36.8%

Hampshire n = 11	45.5%	54.5%	0%	0%

Piétrain n = 16	13.4%	26.6%	33.4%	26.6%

^1) ^RHS of 0.21 to 3.33; ^2) ^RHS of 3.34 to 18.8; ^3) ^RHS of 18.81 to 34.91; ^4) ^RHS of 34.92 to 99.12; ^5) ^Eleven German Landrace pigs died or were euthanized outside of days 4 and 20 and, therefore, were not included.

## Discussion

The intention of this study was to establish a combined quantitative scoring system to determine the lung status of diseased pigs avoiding the necessity of necropsy. All individual examination methods had been established previously, and the resulting individual scores (clinical, ultrasonographic and radiographic score) had been evaluated [31,33-36]. However, none of these scores resulted in a ranking of pigs that correlated with the LLS considered as "gold standard". In the current work we examined the suitability of a combined score designated as Respiratory Health Score (RHS) including the clinical score and both ultrasonographic and radiographic examination of the lungs, as lung tissue alterations do not inevitably lead to clinical symptoms. These lung tissue alterations, however, can be visualized using imaging techniques with both radiographic and ultrasonographic examination methods having different limitations [37-42]. Also, neither ultrasonography nor radiography alone accurately depict the severity of lung tissue alterations. While affections of the pleura as well as sequestration of lung tissue at the lung surface could be clearly identified during the ultrasonographic examination, deep tissue alterations with no contact to the lung surface could only be detected radiographically. Using both methods, however, allows a comprehensive evaluation of the lung condition [31]. For this reason the results of both methods are combined with the clinical score to result in a single value, the RHS. The RHS and the LLS are significantly correlated and, therefore, the RHS was considered appropriate as an alternative to the LLS. Since both imaging techniques can only be performed under anaesthesia the frequency of investigations is limited as immunosuppressive effects from anaesthesia cannot be excluded. In our experimental design we kept at least 14 days between two subsequent anaesthesias thereby minimizing possible detrimental effects. Despite these limitations the RHS, in our hands, is an excellent tool for all scientific investigations, where consecutive quantifications of the lung status during the course of disease in individual pigs are required.

The RHS system was applied to investigate the ranking of pigs in acute (day 4) and chronic infection (day 20 post infection). This comparison of RHS in the acute and chronic stages of disease was done to investigate the impact of the acute lung damage on the course of disease upon *A. pleuropneumoniae *infection. In the past, pigs with a low adaptive immune response have been compared to those with a high adaptive immune response [43]; the latter were then selected for breeding [44] hypothesising that these pigs might show an increase in general resistance to infectious diseases. However, since the degree of acute lung damage which is modulated by the innate immune system appears to have a strong impact on the course of disease the innate immune system might also play an important role in disease development.

Investigating possible differences among the breeding lines used in the study, various environmental factors with a potential influence on susceptibility such as origin of the pigs, age, and infectious dose were all standardized. Sex as another potential factor had been shown previously to not influence the course of disease [24]. Other factors such as the social hierarchy within groups, differences in physical development between breeds [24], and the ability to adapt to new environments (e.g. unfamiliar management procedures or facilities, climate, herd size, or the contact to ubiquitous and contaminating microorganisms) were minimized by the experimental set-up in this study [45,24,46]. Thus, environmental and management conditions were highly standardized, and experiments were performed in groups containing a single breed as well as in groups containing different breeds.

The result of this study confirmed the low susceptibility of Hampshire pigs that had been described previously [24]. Likewise, the previously described susceptibility within the breeds of Large White and German Landrace [23] was confirmed, and pigs of the Piétrain breeding line were found to be equally susceptible based on the RHS. The greater susceptibility of Large White pigs compared to the German Landrace pigs that was described after bacterial lung infections as well as after PCV-2 infections by other workers [23,18] could not be observed. In contrast, the German Landrace pigs have to be considered as most susceptible as only pigs of this breeding line died or had to be euthanized due to severe clinical symptoms. This difference may be due to differences between local breeding lines or simply caused by the small number of Large White pigs in this study.

The genetic mechanisms responsible for the differences observed in susceptibility to *A. pleuropneumoniae *serotype 7 infection are still unknown; since the general mechanisms of pathogenicity are species-specific we expect similar results upon infection with other *A. pleuropneumoniae *serotypes. Future studies on this subject will be greatly facilitated by the finding that the acute lung damage appears to be decisive for the course of disease which allows termination of infection experiments on day 4 post infection without loss of information. Therefore, this work is considered to be an essential first step in the development of genetic markers that could be used for a breeding selection aimed at the increase of resistance to *A. pleuropneumoniae *infection. In addition, it might facilitate studies investigating genetic resistance of pigs to other bacterial respiratory tract pathogens.

## Conclusion

In the course of these studies we demonstrated that the Respiratory Health Score (RHS) developed shows a highly significant correlation to the Lung Lesion Score (LLS) considered as a "gold standard". Therefore, the RHS allows the comparison of the lung status at different time points during infection in the same pig. The results of the lung status comparison between acute and chronic stage of pleuropneumonia strongly suggests that the acute lung damage is decisive for the course of disease. Furthermore, the different severity of symptoms among the tested breeding lines clearly implies that they have a highly distinct genetic susceptibility to *A. pleuropneumoniae *infection.

## Methods

### Animals, animal housing, and time course

A total of 106 pigs (40 German Landrace, 28 Pietrain, 22 Hampshire, 16 Large White) were infected. Three pigs (two German Landrace, one Hampshire) were not included into the study since the had contracted Oedema Disease. Due to severe clinical symptoms seven animals died or were sacrificed before day 4, and four animals were sacrificed after day 4 and before day 20 (all German Landrace). Thus, for 92 pigs (27 German Landrace, 28 Piétrain, 21 Hampshire, 16 Large White) both, a RHS and a LLS could be obtained. Of these, 47 pigs (9 German Landrace, 12 Piétrain, 10 Hampshire, 16 Large White) were randomly selected and sacrificed on day 4; the remaining 45 pigs (18 German Landrace, 16 Piétrain, 11 Hampshire) were sacrificed on day 20 post infection. Material from the same pigs was simultaneously used in a parallel study investigating expression levels of immune markers in relation to breeding line and the disease severity [47].

At the time of infection pigs were 6 to 7 weeks old and were tested negative for *A. pleuropneumoniae *using an ApxIIA [48] and an ApxIVA ELISA [49]. In addition, all pigs tested negative for *Mycoplasma hyopneumoniae *and *PRRS *by antibody ELISA (HerdChek*, IDEXX Laboratories, Westbrook, Maine, USA), and *Influenca-A-Virus *by hemagglutination inhibition test using porcine H1N1, H1N2 and H3N2 strains. During the entire time (before and after infection) pigs were kept under standardized containment level 2 conditions with 8 m^2 ^floor space per 10 pigs, with standardized climate and diet, and cared for in accordance with the principles outlined by the European Convention for the Protection of Vertebrate Animals used for Experimental and Other Scientific Purposes (European Treaty Series, nos. 123  and 170 ; approval number: 33-42502-05/941). In order to reduce the impact of stress on the examination results pigs were randomised into different groups at the age of four weeks and accustomed to handling over a one- to two-week period prior to aerosol infection. The examination period lasted four weeks, from seven days before until 20 days after infection. Radiography and sonography were done three times, namely on day 7 before and on days 4 and 20 after infection.

At the end of the experiment pigs were necropsised; the lung damage was quantified using the Lung Lesion Score (LLS) proposed by Hannan et al. [32] and specified in the European Pharmacopoeia for the testing of *A. pleuropneumoniae *vaccines (3 rd edn., EDQM, Council of Europe, Strassbourg, France). The LLS is determined after the removal of the lungs from the thorax in the course of necropsy. The lungs are palpated and areas of non-physiological consistence are recorded in a schematic map of the porcine lung. In this map the pulmonary lobes are subdivided into triangles (7 triangles for each, cranial and middle lobes, 19 triangles for caudal lobes, and 8 triangles for the Lobus accessories). In the LLS (maximum value of 35) each lobe contributes a maximum score of 5 (tissue damage in the entire lobe). To determine the LLS the damaged area is determined by counting the number of triangles indicating tissue damage and expressing it as a fraction of five for each lobe. The values for each of the seven lobes are added to result in the LLS.

### Experimental infection and clinical investigation

Aerosol infection was performed using *A. pleuropneumoniae *serotype 7 strain AP76 as previously described [50,51]; during infection approximately 1 × 10^5 ^bacteria were nebulised for five pigs resulting in an aerosol with 1 × 10^2 ^colony forming units (cfu) of *A. pleuropneumoniae *per litre aerosol.

Clinical signs were monitored daily and consisted of an assessment of general appearance (posture, behaviour, feed intake, temperature, vomiting) and respiratory tract (breathing noise, dyspnoea, respiratory frequency, coughing, pulsoxymetric oxygen saturation [Pulsoxymeter NPB-40, Fa. Nellcor Puritan Benett Inc., Boulder, U.S.A.] and cyanosis); pulsoxymetric oxygen saturation was measured at the lower side of the tail [33]. Parameters with two specifications (vomiting) were scored with 0 or 4 points, parameters with three specifications (dyspnoe) with 0, 2, or 4 points, parameters with four specifications (breathing noise, coughing, feed intake) with 0, 1.33, 2,67, or 4 points, and the remaining parameters with five specifications with 0, 1, 2, 3, or 4 points [31]. The points were added and divided by 11 to result in the daily clinical score resulting in a score of 0 for a healthy animal and a maximum score of 4 for a severely diseased animal; a dead animal was scored with 5 points. The clinical score on day 4 is the sum of the daily clinical score from days 1 to 4; the clinical score on day 20 is calculated accordingly [31].

Ultrasonographic and radiographic examinations of the lungs both were performed under anaesthesia with 15 mg/kg Ketamine i.m. (Ursotamin^®^, Fa. Serum-Werk-Bernburg AG, Bernburg, Germany) and 2 mg/kg i.m. Azaperon (Stresnil^®^, Fa. Janssen-Cilag GmbH, Baar, Switzerland). Ultrasonography of the lungs was performed with an 8 MHz linear scanner (LOGIQ™ Book XP, Fa. GE Medical Systems, Chalfont St. Giles, Great Britain) in lateral position. On either side of the chest each intercostal space was scanned in dorso-ventral direction. Right and left lung are each divided into 5 sections, and each section is sub-divided into the intercostal spaces [31]. Comet-tail-artefacts, echogenicity, consolidations, and sequesters were analysed with a point range from 0 (physiological findings) to 8 (no unaltered lung tissue seen). The addition of the score points given for the observed alterations resulted in the sonographic score with a score of 200 being lethal [31].

Radiography was carried out in two views (latero-lateral and dorso-ventral; Convix Generator 360, Fa. Picker Int., Munich, Germany, 400 mAs, 110 kV, 1000 ms) using a shutter priority (Precimat, Fa. Picker Int.). In the radiographic score the lung was divided into eight sections. Bronchial, alveolar and interstitial patterns, cardiac and diaphragm silhouette and sequesters were analysed with a point range from 0 to 3. The addition of the score points given for the observed alterations resulted in the radiographic score with a score of 50 being lethal [31].

### Development of a Respiratory Health Score (RHS)

For quantification of the examination results the Respiratory Health Score (RHS) was developed based on a combination of the clinical, sonographic and radiographic scores. The RHS is a score that indicates the degree of lung alteration in percent of the maximum alteration possible in a live animal. Briefly, individual scores are normalized by division with the score causing death in an animal, added, divided by three and multiplied by 100 in order to get a value in percent (Fig. 1). Therefore, the RHS has a possible range from 0 to 100%.

### Statistical analysis

For statistical analyses of the data SAS^® ^software (SAS Institute Inc., Cary, NC, USA) was used. Correlations of i) LLS and RHS and ii) RHS on days 4 and day 20 post infection were assessed by calculating the Spearman Rank Correlation Coefficient.

## Authors' contributions

DH participated in the design and carried out the clinical studies, performed the statistical analysis, participated in the development of the Respiratory Health Score and drafted the manuscript. IHP participated in the design of the studies and helped to draft the manuscript. KT participated in the clinical studies. TR organized all clinical studies and carried out the aerosol infection. MB developed the Respiratory Health Score. KSM carried out and interpreted the serological testing of all pigs entering the study. GFG conceived of and designed the studies, participated in their coordination and helped to draft the manuscript. KHW participated in the design and the coordination of the studies.
